# Special Issue “Pathogenesis, Epidemiology and Treatment of Atopic Dermatitis and Psoriasis”

**DOI:** 10.3390/jcm10235701

**Published:** 2021-12-04

**Authors:** Masutaka Furue

**Affiliations:** Department of Dermatology, Kyushu University, Maidashi 3-1-1, Higashiku, Fukuoka 812-8582, Japan; furuemasutaka00@yahoo.co.jp

Atopic dermatitis and psoriasis are common inflammatory skin diseases that enormously deteriorate the psycho-physical and socio-economic condition of the patients who are afflicted with these conditions. Although the epidermal keratinocytes are the major targets, differential immune responses have been found to operate in the pathomechanisms of atopic dermatitis and psoriasis. The recent therapeutic success of specific biologics points a crucial role of IL-13/IL-4 and IL-22 in atopic dermatitis and of TNF-α, IL-23, and IL-17A in psoriasis. These cytokines differentially affect epidermal barrier function, epidermal proliferation, and the inflammatory response of the skin with variable levels of pruritus and scratch injury. As the skin is constantly exposed to the outside atmosphere, a myriad of external stimuli such as ultraviolet rays and environmental chemicals may exacerbate or ameliorate cytokine-mediated keratinocyte alteration. Thus, the skin barrier, itching, and inflammation are current and future treatment targets for both diseases.

In this Special Issue, Montero-Vilchez et al. showed direct evidence of skin barrier dysfunction in psoriasis and atopic dermatitis [1]. The IL-13/IL-4 axis plays a major and pathogenic role in atopic dermatitis because its overdrive downregulates the expression of barrier-related molecules such as filaggrin. The overdrive of the IL-13/IL-4 axis can be treated by steroid, tacrolimus, cyclosporine, and Janus kinase inhibitors. The anti-IL-4 receptor α antibody dupilumab interferes with IL-13/IL-4 binding [2]. The aryl hydrocarbon receptor (AHR) axis counteracts the IL-13/IL-4 axis and is able to normalize the IL-13/IL-4-mediated decrease of barrier-related proteins. Some antioxidative AHR agonists such as coal tar and glyteer are used as traditional remedies. Recently, another antioxidative AHR agonist tapinarof began clinical trials [2]. The topical application of a cosmetic AHR agonist may be also useful for the maintenance of healthy skin [3].

IL-31 has gained particular attention in the discussion of skin inflammation because it induces non-histaminergic pruritus. The anti-IL-31 receptor antibody nemolizumab is known to reduce the itch sensation in atopic dermatitis and other pruritic skin conditions [4]. The overproduction of IL-13/IL-4 is induced by the excessive activation of type 2 helper T (Th2) cells. OX40/OX40L signaling plays a critical role in the full activation of Th2 cells. Therefore, targeting the OX40/OX40L axis is the new strategy for treating atopic dermatitis [5].

Recent advances in skin biology are leading us towards solving the enigma of complex skin inflammation. We are expecting new publications in this Special Issue.
